# Exposure of Small-Scale Gold Miners in Prestea to Mercury, Ghana, 2012

**DOI:** 10.11604/pamj.supp.2016.25.1.6171

**Published:** 2016-10-01

**Authors:** Ebenezer Kofi Mensah, Edwin Afari, Frederick Wurapa, Samuel Sackey, Albert Quainoo, Ernest Kenu, Kofi Mensah Nyarko

**Affiliations:** 1 Ghana Field Epidemiology and Laboratory Training Programme, School of Public Health, University of Ghana, Legon, Ghana; 2 Ghana Health Service; 3 Ghana College of Physicians and Surgeons

**Keywords:** Mercury, retort, personal protective equipment, Prestea, Ghana

## Abstract

**Introduction:**

Small-scale gold miners in Ghana have been using mercury to amalgamate gold for many years. Mercury is toxic even at low concentration. We assessed occupational exposure of small-scale gold miners to mercury in Prestea, a gold mining town in Ghana**.**

**Methods:**

We conducted a cross-sectional study in which we collected morning urine samples from 343 small-scale gold miners and tested for elemental mercury. Data on small-scale gold miner's socio-demographics, adverse health effects and occupational factors for mercury exposure were obtained and analyzed using SPSS Version 16 to determine frequency and percentage. Bivariate analysis was used to determine occupational factors associated with mercury exposure at 95% confidence level.

**Results:**

The mean age of the small-scale gold miners was 29.5 ±9.6 years, and 323(94.20%) were males. One hundred and sixty (46.65%) of the small-scale gold miners had urine mercury above the recommended exposure limit (<5.0ug/L). Complaints of numbness were significantly associated with mercury exposure among those who have previously worked at other small-scale gold mines (χ^2^=4.96, p=0.03). The use of personal protective equipment among the small-scale gold miners was low. Retorts, which are globally recommended for burning amalgam, were not found at mining sites.

**Conclusion:**

A large proportion of small-scale gold miners in Prestea were having mercury exposure in excess of occupational exposure limits, and are at risk of experiencing adverse health related complications. Ghana Environmental Protection Agency should organize training for the miners.

## Introduction

Mercury is a chemical element commonly used in small-scale gold mining to amalgamate gold. It is estimated that there are between 10-15 million small-scale gold miners worldwide [1]. The small-scale gold mining sector contributes about 25% to total global gold production [2]. Small-scale gold mining serves as an important source of livelihood for many rural communities, and is also the world's fastest source of mercury contamination[3]. Mercury has effects on the nervous system as was seen in human poisoning in Japan and Iraq [4] and is toxic even at low concentrations [5]. Over one million Ghanaians are estimated to be working in the small-scale gold mining operations[6]. The bulk of small-scale gold mining is concentrated in the Western part of Ghana especially Prestea which is an old gold mining town. Small-scale gold mining activities in Ghana using mercury creates environmental problems such as land degradation, loss of natural resources and water pollution [7]. Small-scale gold miners in Prestea, Ghana handle mercury without appropriate respiratory and skin protection. In Ghana, small-scale gold miners add mercury to the gold containing ore and mix the two metals by hand without gloves to form an amalgam[8]. Subsequent burning of this amalgam over a charcoal fire releases mercury vapour. The released mercury vapour poses both an occupational and environmental threat, and those involved in small-scale gold mining activities as well as residents may inhale high levels of elemental mercury vapour. A study done in the Talensi-Nabdam District in the Upper East Region of Ghana, in 2009 reported high levels of urine mercury among small-scale gold miners [9]. Another study also found low urine mercury levels among small-scale gold miners in Dunkwa-on-Offin, in the Central Region of Ghana [10]. Small-scale gold miners in Prestea are at risk of mercury exposure, and there is paucity of information on their occupational exposure to mercury. We assessed occupational exposure of small-scale gold miners in Prestea to mercury.

## Methods

**Study design and setting:** we conducted a cross sectional study among small-scale gold miners in Prestea. Prestea is in the PresteaHuni-Valley District of the Western Region of Ghana. Small-scale gold mining activities are widespread in the town employing more than 1000 people with some migrating from other towns in Ghana.

**Participant selection:** a minimum sample size of 334 was obtained; using a prevalence of 68% mercury exposure among small-scale gold miners in South-Western Ghana [11], and an allowable error of 5% at 95% confidence level. Three hundred and forty-three small scale gold miners were recruited into the study. Twenty out of the 25 small-scale gold mining sites scattered in Prestea were randomly selected. At each mining site, small scale gold miners were selected randomly, the total number selected per mining site was based on probability proportional to size.

**Data collection:** a pre-tested semi-structured questionnaire was administered to the small-scale gold miners to document their socio-demographics characteristics, occupational exposure and safety, and presence of signs and symptoms suggestive of mercury exposure. Work place assessments through direct observation and interviews were conducted at the small-scale gold mining sites and shops where amalgam burning takes place to assess occupational exposure.

**Urine collection and transportation:** we collected 30ml-50ml morning urine samples from each small-scale gold miner into polyethylene mercury free plastic bottles. All urine samples were kept in cold boxes containing ice packs with a thermometer to maintain a temperature between 2oC to 8oC in the field, and returned to a fridge at Prestea Government Hospital Laboratory each day at the same temperature range. For every 30ml of urine,2ml of 5% volume by volume Nitric acid was added to digest mercury into its inorganic forms. To ensure anonymity, all urine containers bore only the identifying numbers of the small scale gold miners. Urine samples maintained at a temperature of 2oC to 8oC in cold boxes were sent to the Water Research Institute Laboratory in Accra, for mercury analysis.

**Urine mercury determination:** the Cold Vapour Atomic Absorption Spectrophotometer machine model AA240F was used for the urine mercury analysis. Urine mercury determinations were calibrated to obtain a calibration curve by preparing three mercury standards (10ug/L, 20ug/L and 50ug/L) from a commercially prepared mercury stock standard of 1000ppm in 0.5M Nitric acid. These calibration standards were prepared fresh for each batch of 25 urine samples run daily; and run in parallel with the test urine samples and a commercially prepared quality control mercury sample. We defined occupational mercury exposure as urine sample containing mercury ≥5.0ug/L[12].

**Data management and analysis:** data were coded, entered and analyzed using Statistical Package for Social Sciences(SPSS)Windows version 16. we summarised continuous variables as means and SD and categorical variable as proportions, and presented the results in graphs and tables. Pearson's Chi-square test was used to determine association between mercury exposure and any adverse health effects at 95% confidence level. Bivariate analysis was done to determine occupational factors associated with mercury exposure and to generate prevalence odd ratios (OR) at 95% confidence level.

**Ethical Consideration:** ethical clearance was obtained from Ghana Health Service Ethics Review Comittee. We obtained informed consent from the small-scale gold miners.

## Results

**Socio-demographic characteristics:** three hundred and forty three small-scale gold miners were recruited in this study. The ages of the small-scale gold miners ranged from 15-70 years with a mean age of 29.46 ±9.64 years. Majority, 154/343(44.9%), of the small-scale gold miners were within the age 20-29 years, and 323/343(94.2%) were males. Their duration of work ranged from 1-38 years with a mean occupational exposure of 7.19 ± 6.96 years. Two hundred and nine, (60.9%) had primary education (Table 1).

**Table 1:** Demographic characteristics of small scale gold miners, Prestea, Ghana, 2012

Variables	Frequency (N=343)	Percentage (%)
**Sex**		
Male	323	94.2
Female	20	5.8
**Age group (years)**	-	-
<19	44	12.8
20-29	154	44.9
30-39	88	25.7
40-49	48	14.0
50-59	5	1.5
>60	4	1.2
**Educational level**	-	-
None	33	9.6
Primary	209	60.9
Secondary	88	25.7
Tertiary	13	3.8

**Figure 1:**
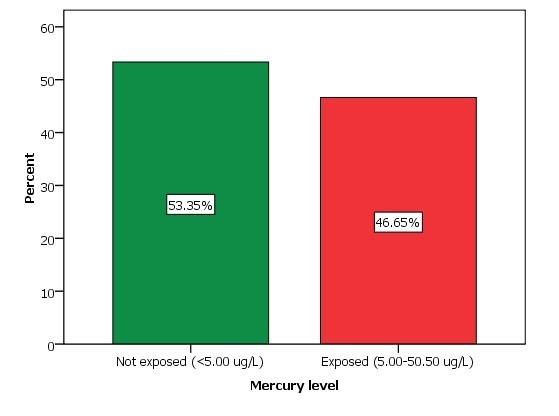
Distribution of mercury exposure among small-scale gold miners, Prestea, Ghana, 2012

**Mercury exposure:** the study found 160 small-scale gold miners (46.65%) who were exposed to mercury. The exposure level range was between (5.00-50.50ug/L) of mercury with a mean of 14.75 ± 10.22ug/L(Figure 1).

**Signs and symptoms suggestive of mercury exposure:** an association was found between mercury exposure and complaints of skin rashes among the 343 small-scale gold miners; however, this finding (Table 2) was not statistically significant(χ^2^=3.49, p=0.062). Twenty-five out of the 343 small scale gold miners reported working previously at other small scale gold mining sites before migrating to work in Prestea. Among them complaints of numbness was significantly associated with mercury exposure (χ^2^=4.96, p=0.026) (Table 2). There was no statistically significant association between exposure to mercury and complaint of red eyes (χ^2^=3.22, p=0.073), medical taste (χ^2^=3.72, p=0.054) among the 25 small-scale gold miners (Table 2).

**Table 2:** Association between Signs and symptoms; and mercury exposure among small scale gold miners, Prestea, Ghana, 2012

Signs and symptoms	N=343		N=25	
	χ^2^	p-value	χ^2^	p-value
Red eyes	0.26	0.608	3.22	0.073
Skin rashes	3.49	0.062	0.33	0.566
Frequent cough	0.23	0.629	0.37	0.546
Persistent fever	0.07	0.788	0.37	0.539
Persistent headache	0.69	0.405	0.67	0.412
Metallic taste	0.37	0.544	3.72	0.054
Fatigue	0.01	0.930	1.97	0.160
Muscle aches	0.14	0.706	0.11	0.739
Sinusitis	0.40	0.525	0.00	0.959
Insomnia	0.96	0.327	2.39	0.122
Numbness	0.00	0.959	4.96	0.026
Hair loss	0.04	0.839	0.81	0.369

**Knowledge about mercury hazards and the use of personal protective equipment:** two hundred and thirty-six (68.8%) store mercury at home and 225(65.6%) had no knowledge about hazards associated with mercury use. Majority, 335(97.7%) had no occupational safety training in handling mercury. The use of personal protective equipment (PPE) among the small-scale gold miners was limited: 336(98.0%) did not use aprons, 315(91.8%) did not use face marks, 321(93.6%) did not use rubber gloves, 310(90.4%) did not use leather boots, and 314(91.5%) did not use head coverings, in their work. Some of the small-scale gold miners during this study were seen removing excess mercury from amalgam with their bare hands without gloves; whereas others sucked the mercury from the amalgam with their mouth for re-use.

**Occupational risk factors associated with mercury exposure at bivariate analysis:** none of the occupational risk factors in the small-scale gold mining was associated with high mercury exposure (Table 3).

**Table 3:** Bivariate analysis of occupational factors associated with mercury exposure among small scale gold miners, Prestea, Ghana, 2012

Occupational Factors	OR(95%C.I)	p-value
Amalgamating with mercury	1.7(0.9,3.2)	0.095
Burning of Amalgam	1.3(0.7,2.4)	0.436
Smelting of Gold	1.0(0.7,1.6)	0.898
Transporting of Mercury	1.4(0.8,2.4)	0.257
Transporting of ore	1.1(0.8,1.8)	0.521
Standing in a pool of water/stream whiles working	1.4(0.9,2.2)	0.134
Sucking excess mercury from the amalgam for re-use	1.5(0.9,2,3)	0.100

## Discussion

This study found 46.65% of the small-scale gold miners occupationally exposed to mercury (≥5.0ug/L)[12]. This finding is similar to studies conducted in South Africa, Venezuela and Brazil where estimated urine mercury concentrations among small-scale gold miners were 50%, 48.3% and 52% respectively above the recommended occupational exposure limits[12–14]. A study in Burkina Faso, however, reported 69% of small-scale gold miners having urine mercury levels above the recommended occupational exposure limit [15]. The findings in this present study show that small-scale gold miners living in Prestea are potentially exposed to mercury. These small-scale gold miners are at risk of experiencing adverse health effects and mercury intoxication. Complaints of numbness were significantly associated with mercury exposure among small-scale gold miners who have previously worked at other small-scale gold mining sites before migrating to work in Prestea. This could be attributed to continuous, higher and longer period of occupational mercury exposure among this group of small-scale miners. Similar studies conducted in Ghana and Venezuela did not find any significant association between mercury exposure and frequency of reported symptoms of mercury among small-scale gold miners[9, 13]. A recent study conducted in Burkina Faso found small-scale gold miners directly involved in gold mining activities reporting symptoms relating to mercury poisoning [15]. The low level education (primary) among the small-scale gold miners could explain the limited use of PPE among the study population. This low level use of PPE is consistent with other findings in Venezuela and Ghana [13, 16]. Other possible explanation is their lack of knowledge and apathy towards mercury exposure. Our study did not find any significant association between occupational risk factors and mercury exposure; this is contrary to a study done in the Talensi-Nabdam District in the Upper East Region of Ghana where amalgam burners were found to have significantly higher urine mercury [9]. These study subjects may have been exposed to relatively higher and longer period of mercury vapour compared to those in this study. The inappropriate method of sucking mercury with the mouth, and the use of bare hands to squeeze mercury from the amalgam for re-use, could lead to mercury accumulating in their bodies causing symptoms such as dermatitis, cutaneous eruptions and numbness. Retorts which are globally recommended for burning gold amalgam [17] were not found at small-scale gold mining sites visited. However, a study done in Tanzania found some small-scale gold miners using retorts[18]. Studies have shown that it is possible to reduce mercury exposure at small-scale gold mining sites by using retorts [19, 20].

**Limitation:** Due to the cross-sectional study design, we were unable to determine pre-occupational exposure to mercury before urine collection. Signs were elicited and symptoms self-reported; and these are subjective.

## Conclusion

Some small-scale gold miners in Prestea were exposed to mercury levels in excess of occupational exposure limits. Complains of numbness, metallic tastes, skin rashes and red eyes were elicited and reported by some small scale gold miners which might be related to mercury exposure. Environmental Protection Agency (EPA) of Ghana should organize training programmes in safety and hazards associated with handling of mercury among small-scale gold miners in Prestea to raise awareness concerning mercury exposure as well as ensuring that these PPEs are used appropriately. The District field officers of EPA should regularly monitor and supervise the activities of these small-scale gold miners to ensure that the environment is not destroyed through indiscriminate use of mercury. Ghana's Minerals Commission should contract local craft manufacturers to produce retorts at affordable prices for sale to the small-scale gold miners. They should also identify safe designated areas for the small-scale gold miners especially those working near human settlements in order to minimize environmental degradation as well as human exposure due to mercury use.
